# Nrf2 Activation in Chronic Kidney Disease: Promises and Pitfalls

**DOI:** 10.3390/antiox11061112

**Published:** 2022-06-03

**Authors:** Ana Karina Aranda-Rivera, Alfredo Cruz-Gregorio, José Pedraza-Chaverri, Alexandra Scholze

**Affiliations:** 1 Laboratory F-315, Department of Biology, Faculty of Chemistry, National Autonomous University of Mexico, Mexico City 04510, Mexico; anaaranda025@gmail.com (A.K.A.-R.); cruzgalfredo@gmail.com (A.C.-G.); pedraza@unam.mx (J.P.-C.); 2 Department of Nephrology, Odense University Hospital, 5000 Odense C, Denmark; 3 Institute of Clinical Research, University of Southern Denmark, 5000 Odense C, Denmark

**Keywords:** Nrf2, oxidative stress, CKD, bardoxolone methyl, fibrosis, inflammation, NQO1, kidney function, hemodialysis, curcumin, redox signaling

## Abstract

The nuclear factor erythroid 2-related factor 2 (Nrf2) protects the cell against oxidative damage. The Nrf2 system comprises a complex network that functions to ensure adequate responses to redox perturbations, but also metabolic demands and cellular stresses. It must be kept within a physiologic activity range. Oxidative stress and alterations in Nrf2-system activity are central for chronic-kidney-disease (CKD) progression and CKD-related morbidity. Activation of the Nrf2 system in CKD is in multiple ways related to inflammation, kidney fibrosis, and mitochondrial and metabolic effects. In human CKD, both endogenous Nrf2 activation and repression exist. The state of the Nrf2 system varies with the cause of kidney disease, comorbidities, stage of CKD, and severity of uremic toxin accumulation and inflammation. An earlier CKD stage, rapid progression of kidney disease, and inflammatory processes are associated with more robust Nrf2-system activation. Advanced CKD is associated with stronger Nrf2-system repression. Nrf2 activation is related to oxidative stress and moderate uremic toxin and nuclear factor kappa B (NF-κB) elevations. Nrf2 repression relates to high uremic toxin and NF-κB concentrations, and may be related to Kelch-like ECH-associated protein 1 (Keap1)-independent Nrf2 degradation. Furthermore, we review the effects of pharmacological Nrf2 activation by bardoxolone methyl, curcumin, and resveratrol in human CKD and outline strategies for how to adapt future Nrf2-targeted therapies to the requirements of patients with CKD.

## 1. Introduction to Chronic Kidney Disease

Chronic kidney disease (CKD) comprises a heterogeneous group of kidney disorders and is defined by alterations in kidney function or structure for more than three months. CKD is classified and staged based on the underlying pathology, albuminuria category, and glomerular filtration rate (GFR) [1]. It represents a global public-health problem with a major effect on global morbidity and mortality. Better strategies for early detection and prevention of CKD as well as new effective therapies are needed. [2]. Factors that are associated with CKD progression include the cause of CKD, category of GFR and albuminuria, age, elevation of blood pressure, and history of cardiovascular disease (CVD) [1]. Inflammatory processes and oxidative damage are important pathomechanisms for CKD progression and CKD-attributable morbidity, such as CKD-associated early vascular aging [3,4], impaired immune function [5,6] or anemia [7,8]. An overview of all abbreviations used in the article is provided as Supplementary Table S1 online.

## 2. Introduction to the Nrf2 Pathway

**Figure 1:**
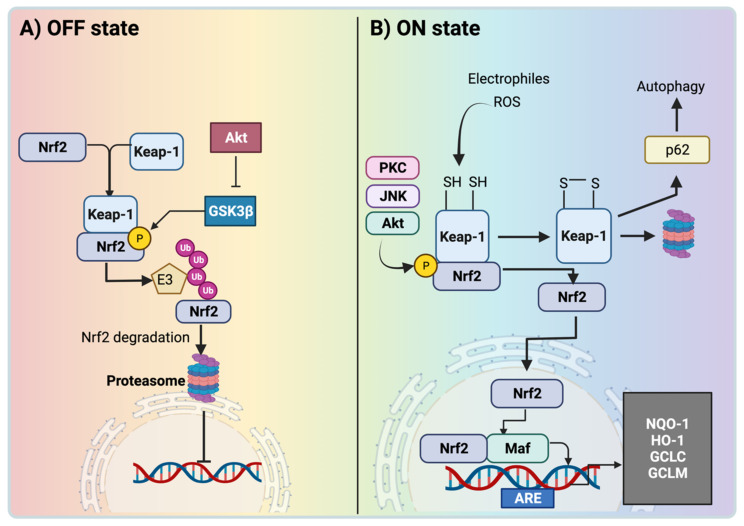
Nuclear factor erythroid 2-related factor 2. (**A**) In the OFF state, the nuclear factor erythroid 2-related factor 2 (Nrf2) is kept at a basal activity level by Kelch-like ECH-associated protein 1 (Keap1). In addition, the inhibition of Akt induces the upregulation of glycogen synthase kinase 3 beta (GSK3β), promoting the phosphorylation and degradation of Nrf2. Both mechanisms lead to Nrf2 degradation via the proteasome. (**B**) In the ON state, electrophiles and ROS modify the cysteine residues of Keap1, promoting its degradation via proteasome or via sequestosome (p62). The latter induces the nuclear translocation of Nrf2 to interact with the musculoaponeurotic fibrosarcoma (maf) proteins and bind to antioxidant response elements (AREs), triggering the expression of NADPH quinone oxidoreductase (NQO1), heme oxygenase-1 (HO-1), glutamate-cysteine ligase catalytic (GCLC), and glutamate-cysteine ligase modifier (GCLM). The figure was created using BioRender.

Oxidative stress is one of the principal contributors to CKD development. CKD presents upregulation of NADPH oxidases and mitochondria dysfunction, favoring reactive-oxygen-species (ROS) overproduction. Additionally, antioxidant mechanisms are reduced, preventing an effective ROS decrease and causing oxidative stress. On the other hand, ROS activate transcription factors to decrease it [9], which is fundamental to alleviating oxidative stress in CKD. Note that cells maintain a redox state (balance between ROS and antioxidants) by activating different transcription factors that induce the transcription of different antioxidant enzymes to protect against oxidative stress [10,11]. Nuclear factor erythroid 2-related factor 2 (Nrf2) is a ubiquitous protein that contains 605 amino acids, which modulate oxidative stress in the cells by functioning as a transcription factor that responds to ROS overproduction. This basic leucine zipper (bZip) transcription factor binds to DNA in an acidic region, regulating its transcriptional activity [12]. A negative regulator of Nrf2 is Kelch-like ECH-associated protein 1 (Keap1), a protein that mediates Nrf2 degradation via the proteasome. This process requires the bridge formation between Nrf2 and the complex E3 ubiquitin ligase, constituted by the cullin3 (Cul3)-ring box 1 (Rbx1). Nrf2 can also be degraded via proteasome independently of Keap1. The latter mechanism involves F-box/WD repeat-containing protein 1A, known as β-TrCP, and glycogen synthase kinase 3 (GSK3) [13,14]. Under normal conditions, the activity of GSK3 is inhibited by protein kinase B, better known as Akt; however, the inactivation of Akt induces the phosphorylation of Nrf2 via GSK3β, promoting Nrf2 recruitment and proteasomal degradation via β-TrCP. Other kinases can regulate Nrf2 activity, including AMPK (5’ AMP-activated protein kinase), MAPKs (mitogen-activated protein kinases) family members, and the mechanistic target of rapamycin complex 1 [15] (Figure 1).

Under oxidative-stress conditions, Keap1 is oxidized by electrophiles and oxidants in its cysteine (Cys) residues—Cys 226, Cys 613, and Cys 624—triggering conformational changes that include intramolecular disulfide bridges, permitting Nrf2 release [16]. Then, Keap1 is degraded via the proteasome, allowing Nrf2 accumulation and translocation to the nucleus to interact with musculoaponeurotic fibrosarcoma (Maf) proteins, inducing their association with the 5′-RTGACNNNGC-3′ sequence, known as the antioxidant response element (ARE) in the DNA [17]. An alternative mechanism that mediates Keap1 degradation was described by Taguchi et al. [18]. This mechanism involves the association of the sequestosome (p62) with Keap1, activating autophagy, and in this way, the integrity of the Keap1–Nrf2 system is maintained by sustaining Keap1 turnover [18] (Figure 1).

In the nucleus, Nrf2 modulates the expression of antioxidant and detoxifying enzymes, including glutamate-cysteine ligase modifier (GCLM) subunits, heme oxygenase (HO-1), NADPH dehydrogenase quinone 1 (NQO1) [19,20], glutathione S-transferase (GST), glutathione peroxidase (GPx), and glutamate-cysteine ligase catalytic (GCLC) (Figure 1).

Several reports have attributed that CKD development is deeply associated with oxidative stress, where Nrf2 inactivation is crucial in this process. In the following sections, we discuss the preclinical mechanisms related to Nrf2 activation in CKD, the beneficial effects, the ambiguities, and the disadvantageous effects. We divided these studies into inflammatory, fibrosis, and mitochondrial effects. We also included the effect of Nrf2 in non-CKD models. An overview of the literature search strategy and criteria for study inclusion is provided in the Supplementary Material S1 online.

## 3. Preclinical Data of Salutary, Ambiguous, or Disadvantageous Effects of Nrf2 Activation in CKD

In the kidney, physiological mechanisms such as nitrosylation and glutathionylation depend on ROS; thus, low ROS concentrations are required to maintain redox homeostasis [21,22]. In CKD, NOXs (NADPH oxidases) and mitochondria are the principal renal ROS sources that trigger ROS overproduction, leading to Nrf2 activation [23]. However, in animal models such as diabetic nephropathy (DN), Nrf2 is found to be downregulated, leading to a poor response to ROS overproduction, which in turn promotes oxidative stress. Additionally, it has been shown that Keap1 levels are maintained upregulated, suggesting constant Nrf2 degradation, thereby promoting its downregulation [24].

In contrast, other DN models showed Nrf2 upregulation, but antioxidant response decreased. The latter has been attributed to the inability of Nrf2 to translocate to the nucleus [25]. For instance, the use of epigallocatechin-3-gallate, a green-tea catechin, induced Nrf2 nuclear translocation in streptozotocin-induced DN, which is a condition in which Nrf2 translocation was insufficient [25]. The impairment of Nrf2 nuclear translocation has also been observed in the unilateral-ureteral-obstruction (UUO) model. A study showed that Nrf2 cytoplasmic accumulation was mediated by mitochondrial ROS overproduction, but Nrf2 did not increase in the nucleus, which strongly suggests that Nrf2 nuclear translocation was abolished [26].

The relevance of Nrf2 in CKD is also sustained in the knockout of Nrf2 (Nrf2^−/−^). In these experimental animal models, kidney function was compromised, characterized by lobular formation, cellular proliferation, crescent formation, and subepithelial electron-dense deposits, developing severe lupus-like autoimmune nephritis [27]. Therefore, the activation of Nrf2 has been related to the decrease in ROS overproduction, conferring protection against renal damage [19,28]. The Nrf2-activation-induced renal protection is promoted by using agents or molecules that enhance Nrf2 activity [25,29]. For instance, sulforaphane (SFN), an isothiocyanate derived from cruciferous green vegetables, has been shown to activate Nrf2 by reversibly modifying Keap1 cysteine residues [30]. In a diabetic Nrf2^−/−^ model, NQO1 and HO-1 were downregulated, which increased collagen accumulation in the glomerulus [30]. Moreover, SFN supplementation conferred oxidative DNA damage protection in streptozotocin-induced diabetic nephropathy by promoting the expression of NQO1 and HO-1 [31]. In vitro, the treatment with SFN in human kidney 2 (HK-2) cells decreased ROS derivates such as hydrogen peroxide (H_2_O_2_), which is generated by hippuric acid, promoting the activation of an antioxidant response mediated via Nrf2 [32]. Moreover, in LLCPK (Lilly Laboratories Culture-Porcine Kidney 1) cells treated with cisplatin, SFN protected against oxidative damage induced by cisplatin [33].

In addition to SFN, bardoxolone is known as the most potent Nrf2 activator. Bardoxolone covalently binds to cysteine groups of Keap1 residues, maintaining Nrf2-pathway activation by impeding Keap1 ubiquitination and degradation. The use of RTA 405, a synthetic triterpenoid like bardoxolone methyl, increased GFR in rats. Moreover, in vitro, RTA 405 dose-dependently abolished Ang II-mediated mesangial cell contraction [34]. Although the activation of Nrf2 has beneficial effects, deleterious processes have also been reported. For instance, it was shown that Nrf2 activation induced proteinuria and augmented cardiovascular effects [35,36]. The BEACON study was aborted because the participants in the treatment group showed congestive heart failure [35]. These data suggest that Nrf2 overactivation could be disadvantageous. Furthermore, the overexpression of Nrf2 augmented proteinuria in mice stimulated with Ang II, protein overload, or Adriamycin, causing glomerulosclerosis [37]. In this study, the authors demonstrated that enhancing Nrf2 activation when the damage is established is prejudicial [37]. In addition, the genetic deletion of Keap1 or the pharmacologic Nrf2 activation increases proteinuria levels, causing glomerulosclerosis during pre-existent kidney damage [37].

Furthermore, an experimental assay in mice showed that the deletion of Nrf2 protected against hypertension in diabetic nephropathy [38]. In summary, these data suggest that Nrf2 overactivation could be detrimental to some aspects of CKD. These effects illustrate that if Nrf2 is activated all the time, redox cell-signaling pathways are depleted; therefore, Nrf2 must be kept under homeostatic control within a physiologic activity range [39].

### 3.1. Nrf2 and Inflammation: The Intimate Relation with NF-κB

**Figure 2:**
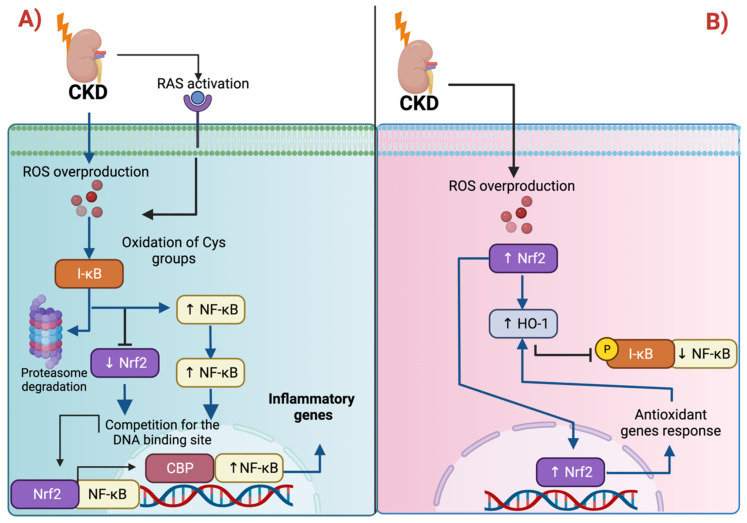
The nuclear factor erythroid 2-related factor 2 (Nrf2) regulates inflammation in chronic kidney disease (CKD). (**A**) The activation of the renin-angiotensin system (RAS) augments ROS production. This ROS oxidizes the cysteine (Cys) groups of the nuclear factor-kappa B (NF-κB) inhibitor, IκB, modifying and promoting its proteasome degradation. This triggers the release of NF-κB, which competes with Nrf2 by the binding DNA site of CREB protein (CBP), (**B**) ROS overproduction also activates Nrf2, inducing its nuclear translocation. The latter upregulates heme oxygenase-1 (HO-1), avoiding NF-κB activation by impeding IκB phosphorylation. The figure was created using BioRender.

Oxidative stress and inflammation are the principal features of CKD development [40]. In the 5/6 nephrectomy model, the inactivation of Nrf2 promoted lipid peroxidation as well as the upregulation of nuclear factor kappa B (NF-κB), the latter leading to monocyte chemoattractant protein-1 (MCP-1) and cyclooxygenase-2 (COX-2) upregulation [41]. Indeed, the downregulation of Nrf2 promotes NF-κB activation because both transcription factors compete by the binding DNA site of cyclic-adenosine-monophosphate (cAMP)-response-element-binding-protein (CREB)-binding protein (CBP), causing the upregulation of NF-κB [42]. In CKD, the activation of the renin-angiotensin system (RAS) generates ROS, stimulating NF-κB to promote the expression of tumor necrosis factor-alpha (TNF-α), interleukin 1 (IL-1), IL-2, and IL-6, MCP-1 (monocyte chemoattractant protein-1), among others [43,44] (Figure 2A). This has been demonstrated because the employment of Nrf2-activating substances decreases NF-κB levels, ameliorating inflammatory kidney markers [45,46]. In line with this, SFN prevents NF-κB activation by impeding IκB phosphorylation and degradation [47]. IκB (nuclear factor of kappa light polypeptide gene enhancer in B-cells inhibitor) is a negative regulator of NF-κB, inhibiting its nuclear translocation; however, the phosphorylation of IκB releases NF-κB, permitting its activation and subsequent nuclear translocation [48]. NF-κB might also be activated via ROS by triggering the proteasomal degradation of IκBα, upregulating inflammatory markers [49]. Thus, ROS regulates NF-κB activity and function.

It has been suggested that targets of Nrf2 control the NF-κB pathway. One of these proteins is HO-1, which blocks the phosphorylation of IκB [50]. HO-1 is a microsomal enzyme that promotes the degradation of the heme group to biliverdin, producing free iron. It has been shown that the production of free iron via HO-1 impedes TNF-α-induced NF-κB activation by preventing the phosphorylation of p65 NF-κB [51]. In CKD, the induction of HO-1 decreases macrophage tubulointerstitial infiltration [52]. Therefore, HO-1 is an essential negative regulator of the NF-κB pathway (Figure 2B).

Conversely, the in vitro upregulation of HO-1 triggered apoptosis in podocytes treated with high glucose levels [53]. In this sense, a study showed that Nrf2 and HO-1 were upregulated in glomeruli of samples of patients suffering from DN, which showed severe ROS overproduction [54]. The same study reported that the deletion of Nrf2 had severe consequences for STZ (streptozocin)-treated Nrf2^−/−^ mice because they had a greater degree of 8-hydroxy-2′-deoxyguanosine (8-Oxo-dG) levels than the STZ-treated Nrf2^+/+^ mice. In untreated conditions, the Nrf2^−/−^ group exhibited more oxidative-stress production, indicating that lacking Nrf2 is deleterious by triggering oxidative damage [54]. Therefore, in DN, the lack of Nrf2 is considered harmful due to the upregulation of oxidative stress, which promotes inflammation due to NF-κB activation.

In the UUO model, monocytes and macrophage infiltration as well as the expression of the inflammatory protein IL-1β are associated with pyroptosis, a type of cell death that involves the inflammation and activation of inflammasomes [55]. The upregulation of NF-κB in CKD is fundamental for promoting the formation of the NOD-like receptor family pyrin domain containing 3 (NLRP3) inflammasome [44,56,57] (Figure 2A). According to the latter, inflammasome assembly requires the activation of NF-κB to trigger the transcription of NLRP3, pro-IL1β, and pro-IL-18, which induces the recruitment of monocytes, macrophages, and lymphocytes [58]. The recruitment of these cells generates additional ROS, promoting a vicious cycle of inflammation and ROS overproduction, which augments renal damage. The activation of Nrf2 has been associated with a decrease in inflammasome components. For instance, in DN, the reduction in NLRP3, ASC, caspase-1, cleaved caspase-1, and IL-1β was attributed to Nrf2 activation [59]. Moreover, the use of isoflavone Biochanin A alleviates fibrosis in the UOO model by inhibiting NF-κB/NLRP3 through Nrf2 activation [60]. On the other hand, in cholesterol crystal-induced atherosclerosis, a possible contributor to CKD-related CVD, Nrf2 activation leads to inflammasome activation, exacerbating atherosclerosis [61]. Collectively, these results show the capacity of Nrf2 and its target proteins to regulate inflammation in CKD through NF-κB. Note that the effects might be context-dependent.

### 3.2. Nrf2 and Fibrosis: The Role of TGF-β1

**Figure 3:**
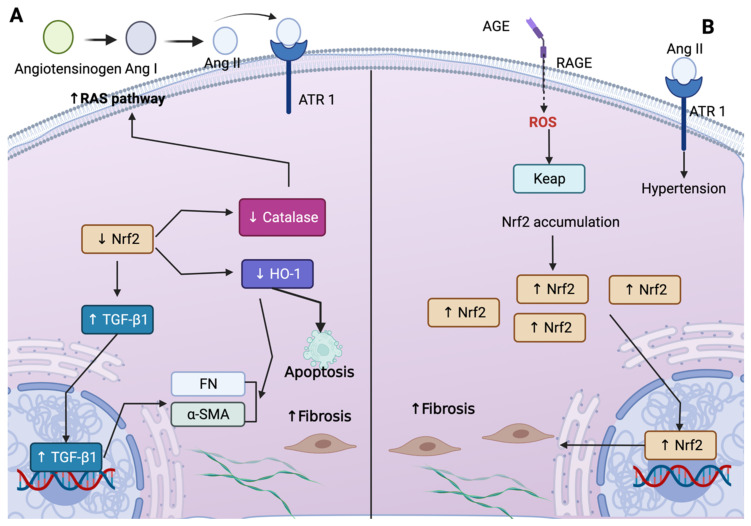
Differing scenarios for nuclear factor erythroid 2-related factor 2 (Nrf2) activation in renal fibrosis. (**A**) The downregulation of Nrf2 (↓Nrf2) induces the upregulation of the transcription growth factor-beta1 (↑TGF-β1), inducing fibrosis by promoting the production of fibronectin (FN) and alpha-smooth muscle actin (α-SMA). In addition, ↓Nrf2 leads to the downregulation of catalase (↓catalase), upregulating the generation of angiotensinogen, which converts into angiotensin II (Ang II). Then, Ang II interacts with angiotensin receptor 1 (ATR1), upregulating the RAS pathway. Nrf2 downregulation also induces the decrease in heme oxygenase-1 (↓HO-1), promoting apoptosis and fibrosis. (**B**) In contrast, the upregulation of Nrf2 (↑Nrf2) induces fibrosis and hypertension. This mechanism is attributed to ROS overproduction, generated via advanced glycation end products (AGEs) by binding to its receptor (RAGE), which activates Nrf2. This mechanism induces Nrf2 translocation to the nucleus with the positioning in Ang/ACE promoter, which leads to fibrosis [38]. The figure was created using BioRender.

Low levels of Nrf2 have been associated with an increase in fibrosis markers. Fibrosis is the common pathway in all CKD forms, independent of the causes [62,63]. A study showed that the deletion of Nrf2 (Nr2^−/−^) in the obstructed kidney induces epithelial–mesenchymal transition (EMT) by promoting the upregulation of fibronectin and alpha-smooth muscle actin (α-SMA) and the downregulation of E-cadherin [64]. In contrast, the induction of Nrf2 was related to the decrease in TGF-β1, the master regulator of fibrosis [60]. Furthermore, the deficiency in HO-1 augmented fibrosis and apoptotic cell death seven days after obstruction [65]. In this study, the authors showed that in mice lacking HO-1, the levels of TGF-β1, α-SMA, and fibronectin were augmented. In contrast, the levels of claudin-2 decreased, suggesting that HO-1 is involved in fibrosis suppression [65]. Furthermore, the overexpression of catalase attenuates fibrosis in diabetic mice, which is attributed to the capability of catalase to decrease ROS and inhibit the RAS pathway [66,67,68]. Accordingly, catalase avoids angiotensinogen mRNA expression, impeding the production of angiotensin I and, later, angiotensin II [66]. In this way, it avoids the binding of angiotensin II to the receptor, the angiotensin receptor 1, impeding the RAS-pathway activation. Thus, Nrf2 and its targets have been suggested to regulate fibrosis through TGF-β1 and RAS pathways (Figure 3A).

Zhao et al. [38] showed that the blocking or deletion of Nrf2 ameliorates tubulointerstitial fibrosis and decreases hypertension and the urine albumin/creatinine ratio. Additionally, in vitro, the silencing of Nrf2 avoided high glucose-induced Nrf2 activation, which prevented the upregulation of angiotensin and angiotensin-converting enzyme (ACE) and the downregulation of angiotensin-converting enzyme 2 (ACE2) [38]. In contrast, Nrf2 wild-type induced ACE and angiotensin (Ang) overexpression. These data suggest that the upregulation of Nrf2 could lead to RAS activation, which generates hypertension in diabetes. According to the authors, the binding of advanced glycation end products (AGEs) produces ROS, which activates Nrf2 by inducing Keap1 cysteine-residue modification, leading to Nrf2 translocation to the nucleus, where it is positioned in the promoter of Ang/ACE gene. The latter upregulates the production of Ang, which might bind to ATR1, activating the RAS-signaling pathway [38] (Figure 3B).

### 3.3. Nrf2 Targets Mitochondrial Proteins

**Figure 4:**
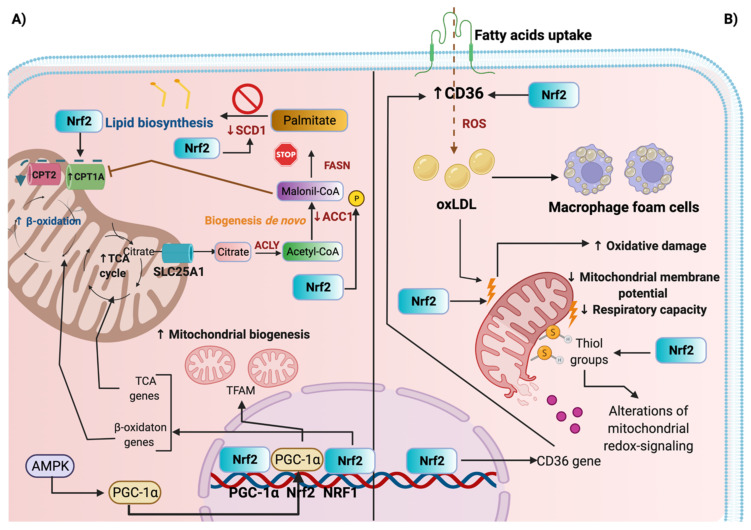
Nuclear factor erythroid 2-related factor 2 (Nrf2) activation and mitochondria. (**A**) Nrf2 upregulates fatty-acid catabolism via β-oxidation, a process occurring in the mitochondria by inducing the expression of carnitine palmitoyltransferase 1 (CPT1), the rate-limiting enzyme in this mechanism. In contrast, Nrf2 inhibits acetyl-CoA carboxylase 1 (ACC1) via phosphorylation, preventing lipid biosynthesis. Furthermore, Nrf2 inhibits stearoyl-CoA desaturase. Nrf2 also induces the expression of the enzymes involved in the tricarboxylic-acid (TCA) cycle, upregulating this pathway. Nrf2 participates in mitochondria biogenesis by interacting with the peroxisome proliferator-activated receptor-gamma coactivator 1-alpha (PGC-1α) and nuclear respiratory factor 1 (NRF1), promoting the expression of transcription factor A, mitochondrial (TFAM). Moreover, NRF1 contains at least four binding sites for Nrf2, participating in antioxidant response. (**B**) Nrf2 overactivation might induce the overexpression of cluster of differentiation 36 (CD36), the principal receptor to fatty-acid uptake, leading to the accumulation of low-density lipoproteins (LDLs) in the cytosol. LDLs are lipids with a major propensity for oxidation, forming oxidized LDLs (oxLDLs), further damaging the mitochondria. Moreover, macrophages engulf oxLDLs, which causes macrophage foam-cell formation. Nrf2 upregulation also induces mitochondrial membrane potential, decreases respiratory capacity, and alters mitochondrial thiol protein groups, leading to the alteration of mitochondrial redox signaling pathways. The figure was created using BioRender.

It has been demonstrated that Nrf2 modulates mitochondrial-protein-induced apoptosis and autophagy [69,70,71]. For instance, in the UUO model, the SFN-mediated Nrf2 activation attenuates ROS production, producing the upregulation of Bcl-2 protein, which avoids apoptosis [55]. In contrast, in the same study, the reduction in the Nrf2 nuclear translocation was attributed to the decrease in Bcl-2 [55]. Moreover, the Nrf2 diminishment is related to the reduction in mitochondrial biogenesis because the nuclear factor respiratory 1 (NRF1) gene promotor, which is an inductor of this process, contains at least four ARE-binding sites for Nrf2 [71,72]. Thus, the downregulation in Nrf2 impacts mitochondrial biogenesis via NRF1. Nrf2 also modulates the expression of mitochondrial proteins involved in mitochondrial biogenesis through the peroxisome proliferator-activated receptor gamma coactivator 1-alpha (PGC-1α) such as TFAM (mitochondrial transcription factor A) [71,73]; however, its mechanism is poorly explored in CKD models. It has been reported that the upregulation of Nrf2 inhibits gluconeogenesis, which is associated with the downregulation of the mRNA expression of PGC-1α [74]. The downregulation of the PGC-1α-induced decrease in mitochondrial biogenesis has been reported in CKD models [75,76,77]. On the other hand, it has been suggested that PGC-1α controls Nrf2 mRNA levels to mediate the antioxidant response [78]. In a premature-kidney-aging model, the mRNA Nrf2 levels were upregulated along with PGC-1α protein levels [79], suggesting an impairment of Nrf2 protein at the translational level. The activation of Nrf2 with SFN augmented Nrf2 but reduced PGC-1α levels, suggesting regulation by decreasing Nrf2 mRNA expression [79]. Indeed, both PGC-1α and Nrf2 established a feedback loop [80]. This feedback loop suggests that Nrf2 might induce PGC-1α activation, and PGC-1α might promote Nrf2 activity (Figure 4A). Therefore, more studies are needed to determine the role of PGC-1α in kidney-aging models.

The downregulation of PGC-1α has deleterious consequences for CKD [76,81,82]. The latter is because PGC-1α regulates the expression of proteins involved in oxidative phosphorylation (OXPHOS), fatty-acid beta-oxidation (β-oxidation), and the tricarboxylic-acid (TCA) cycle; thus, the lessening of PGC-1α might alter these processes. It has been suggested that Nrf2 also controls the expression of proteins involved in these processes such as ATP synthase 5A (ATP5A), cyclooxygenase-4 (COX4), and carnitine palmitoyl transferase-1 (CPT1), among others [83]. Together, these data highlight the involvement of Nrf2 in mitochondrial biogenesis through the regulation of PGC-1α.

Nrf2 might also control fatty-acid β-oxidation, a mechanism occurring in the mitochondria. By this, the use of SFN in type-2-diabetes-induced nephropathy promotes the upregulation of CPT1 and the phosphorylation of acetyl-CoA carboxylase (ACC1), which enhances lipid catabolism [45]. CPT1 is an enzyme involved in the rate-limiting step of β-oxidation by allowing the entry of long fatty acids into mitochondria. On the other hand, the phosphorylation of ACC1 downregulates its activity, decreasing lipid biosynthesis. This study showed that SFN induces AMPK activity, which favors lipid degradation along with the antioxidant response, regulated by Nrf2 [45]. A study showed that other proteins might be positively regulated by Nrf2; however, it is poorly understood in the CKD context [84]. Moreover, the enzymes that catalyze lipid biosynthesis, the stearoyl-CoA desaturase (SCD1) and sterol regulatory element-binding protein-1 (SREBP-1) were downregulated [45]. These data suggest that Nrf2 controlling lipid metabolism is an event present during CKD progression. In line with this, bardoxolone reduces body weight in patients with diabetes, suggesting that proteins that regulate metabolic pathways are targets of Nrf2, and modulating the metabolism might contribute to this mechanism [85].

Bardoxolone use was also shown to impact the mitochondria. A study reported bardoxolone’s adverse effects on the mitochondria of human microvascular endothelial cells-1. In this model, the authors determined that bardoxolone triggers endothelial damage by inducing proton leakage, decreasing mitochondrial membrane potential and respiratory capacity [86]. These mechanisms were attributed to the reactivity of α-cyano-α, β-unsaturated ketone (CUK) moiety in ring A and the ability of bardoxolone to modify thiols of mitochondrial proteins [86], suggesting that the modification of proteins containing thiol groups might be deleterious (Figure 4B). Supporting the latter, the modification of thiol groups showed disadvantages in other studies using electrophiles. For instance, N-acetylcysteine improved the reduced respiratory capacity induced by dimethyl fumarate, an Nrf2 activator, which might suggest that the oxidation of the thiols was the principal action mechanism of dimethyl fumarate [87]. These data suggest Nrf2 overactivation could lead to irreversible modification of thiol groups, altering proteins that are sensitive to redox state located in the mitochondria.

### 3.4. Nrf2 in Non-CKD

The upregulation of Nrf2 in cancer has anticancer proprieties in cancer initiation [88,89]. The latter is because Nrf2 maintains the cellular redox state of the cells, regulating cell proliferation and growth [20]. Note that high levels of ROS maintain the activation of proliferation and cell growth through the activation of signaling pathways such as MAPK kinases [9]. However, when Nrf2 is activated, the antioxidant enzymes targeted by Nrf2 will be induced, reducing ROS and preventing cell proliferation and growth. On the other hand, the inactivation of Nrf2 induces high levels of ROS, which might trigger DNA damage, allowing the appearance of mutations and, in turn, activating the proliferative processes involved in cancer initiation [88]. The cancer-progression stage involves genetic and chromosome instability, causing mutations that permit proliferation and apoptosis evasion [88,90]. Thus, Nrf2 avoids ROS increase, impeding DNA damage, mutations, and genomic stability as hallmarks of cancer and its progression.

However, Nrf2 activation in cancer progression is deleterious because it induces resistance to cancer therapy. For example, in the case of radiotherapy, consisting of ionizing radiation (IR) [91], the generation of high levels of free radicals leads to apoptosis cell death [92]. However, a common consequence of IR is the activation of Nrf2, which triggers a significant tolerance to oxidative stress induced by IR, preventing cancer cell death. Therefore, Nrf2 activation might cause resistance to radiotherapy.

In other models, studies demonstrated that Nrf2 activation induces damage in the myocardium of aging cardiomyocyte-restricted human mutant CryAB transgenic mice [93]. Moreover, in autophagy-deficient mice, Nrf2 accumulation and the upregulation of its target genes were the principal cause of liver injury, suggesting that the upregulation of the Nrf2 pathway might result in deleterious consequences [18]. In atherosclerosis, the Nrf2 upregulation caused free-cholesterol accumulation in macrophages because Nrf2 induces the upregulation of CD36, the principal receptor to fatty-acid uptake [94]. The cholesterol accumulation leads to the formation of macrophage foam cells due to the augmented uptake of oxidized low-density lipoproteins (oxLDL). Indeed, LDLs are highly sensitive to oxidation, being rapidly oxidized under ROS overproduction. OxLDLs might damage the mitochondria, inducing lipid peroxidation (Figure 2B) [95].

In summary, in some contexts the upregulation of Nrf2 might not be beneficial; thus, homeostatic control of Nrf2 is required to avoid fatal consequences.

## 4. Clinical Data of Nrf2 Activation in Human CKD

### 4.1. Endogenous Nrf2 Activation in Human CKD

The transcription factor Nrf2 regulates the gene expression of about 250 target genes involved in redox regulation and antioxidant response, inflammation, heme and iron metabolism, intermediary lipid and carbohydrate metabolism, and reactions of detoxification and biotransformation [96]. An overwhelmingly broad spectrum of factors and cellular conditions that can regulate Nrf2 abundance and activity is known from preclinical studies [97,98,99]. On the contrary, the clinical data on Nrf2 abundance and activity in human CKD are scarce.

#### 4.1.1. Mechanisms Relevant for Endogenous Nrf2 Activation in Human CKD

##### Oxidative Stress

Oxidative stress refers to an imbalance between oxidants and antioxidant mechanisms that leads to deviations from “steady state” redox signaling, and may finally result in oxidative damage of molecules. Pathogenic mechanisms of oxidative stress in CKD have extensively been reported and discussed [100,101,102,103,104,105]. Specific factors, both positive and negative, that influence the overall extent of oxidative stress in human CKD are, for example, related to treatment modality or medication [106,107] and uremic toxin accumulation [108,109,110]. The differential expression of antioxidant and mitochondrial enzymes such as superoxide dismutase 1/2 (SOD1/2), thiosulfate sulfurtransferase (rhodanese), or GPx in dependence of CKD stage or severity is well-described [111,112,113,114]. Activation of Nrf2 in CKD in response or relation to oxidative stress has been reported. In a pharmacological “stress test” study on patients with CKD stage 3 and 4 (CKD3/4), effects of intravenous tin-protoporphyrin application, which transiently induces oxidative stress, were investigated. Around half of those patients suffered from diabetes and three-fourths were hypertensive. The injections resulted in significant increases in plasma concentrations of the Nrf2 target proteins NQO1, HO-1, and ferritin [115]. Interestingly, this study tested the hypothesis that tin-protoporphyrin application could be used to assess the antioxidant reserve in CKD [115]. The response of the three Nrf2 targets was for the most part comparable between CKD3/4 and healthy subjects. This points to a persisting capability in this patient population of CKD3/4 to react to acute oxidative stress via Nrf2 activation, although the cells or tissues responsible for the increased NQO1, HO-1, and ferritin plasma concentrations in this context are not known. In diabetic kidney disease (DKD), an increase in different ROS species has been shown in kidney tissue, as well as mitochondrial fragmentation, seems to be important for hyperglycemia-induced increases in mitochondrial ROS production [102]. Compared to normally functioning kidney-transplant tissue, the activation of Nrf2 was shown in kidney tissue from patients with DKD and proteinuria [54]. In these diabetic glomeruli, Nrf2 and NQO1 protein abundance was increased, and glomeruli showed immunohistochemical signs of oxidative damage. The presence or extent of GFR reduction in these patients was not reported. Likewise, in kidney tissue from patients with Lupus nephritis, who presented with pronounced proteinuria but apparently normal GFR, the glomerular protein amount of Nrf2 and NQO1 was increased compared to healthy kidney tissue, and signs of oxidative damage of the glomeruli were present [116]. Furthermore, in patients with CKD5 and hemodialysis therapy, synovial tissue showed increased HO-1 protein staining together with increased malondialdehyde, the latter an indicator of sustained oxidative stress in this tissue [117].

##### Uremic Toxins

With advancing CKD, an increasing number of substances that normally are eliminated by the kidney accumulate in the body. These uremic toxins contribute to CKD-related complications such as cardiovascular disease and impairment of the immune system. The specific effects of a uremic toxin depend on its nature and concentration.

*Indoxyl sulfate*, a metabolite of the tryptophan pathway, is one of the very important uremic toxins [118]. Indoxyl sulfate (43 mg/L) induced the production of ROS in proximal tubule epithelial cells [109]. In addition, indoxyl sulfate activates the arylhydrocarbon receptor (AhR). Indoxyl-sulfate concentrations of 11 mg/L and 53 mg/L significantly increased TNF-α protein concentration in macrophages through AhR activation [119]. A bi-directional cross-talk between Nrf2 and the AhR exists, and AhR can induce Nrf2 gene transcription [98]. The AhR also induces the expression of the Nrf2 target NQO1 [120]. Therefore, indoxyl sulfate could lead to Nrf2 activation through ROS production and AhR activation. On the other hand, indoxyl sulfate at a concentration of 53 mg/L decreased Nrf2 gene and protein expression in a human proximal tubular cell line [46], and AhR protein was decreased in monocytes of patients with advanced CKD (CKD5 with hemodialysis treatment) compared to healthy control subjects [119]. In human CKD, the relation between indoxyl sulfate and Nrf2 has only sparsely been investigated. The mean indoxyl-sulfate concentration lies around 0.5 mg/L in healthy subjects and ranges from around 5 mg/L in CKD3 to 38 mg/L in CKD5 with hemodialysis treatment (The European Uremic Toxins (EUTox) Database, available online at www.uremic-toxins.org, accessed on 25 February 2022). At indoxyl-sulfate concentrations between 1 mg/L and 11 mg/L in patients with CKD3 and 4, indoxyl sulfate correlated positively with Nrf2 gene expression in peripheral blood polymorphonuclear cells (PBMCs) [121]. In light of a mainly decreased Nrf2 gene expression in advanced CKD [122], a bi-phasic, concentration-dependent effect of indoxyl sulfate in human CKD seems plausible, with Nrf2 activation in the lower concentration range and Nrf2 repression at high concentrations.

*Methylglyoxal* is a uremic toxin that shows around 2.4 times higher concentrations in uremic serum compared to normal serum concentrations (The European Uremic Toxins (EUTox) Database, available online at www.uremic-toxins.org, accessed on 30 March 2022). In human physiology, cellular methylglyoxal is mainly formed through the spontaneous degradation of intermediates of glycolysis. In the cytosol, methylglyoxal is metabolized by glyoxalase 1 [123]. Methylglyoxal that is not metabolized can non-enzymatically modify DNA and proteins. The modification of proteins by methylglyoxal results in protein misfolding and subsequent activation of the unfolded-protein response that has been linked, for example, to the development of DKD [123]. Methylglyoxal has been shown to modify Keap1. This results in cross-linking, with Keap1 dimer formation and resulting Nrf2 accumulation, and an increased expression of the Nrf2 target genes NQO1 and HO-1 [124]. As the intracellular elevation of glucose leads to the accumulation of methylglyoxal, it is likely that methylglyoxal contributes to the increase in Nrf2 and Nrf2 targets NQO1 and HO-1 that has been observed in diabetes without [125,126] and with DKD [37,54,125,126,127,128].

##### Nuclear Factor κ-Light-Chain Enhancer of Activated B Cells (NF-κB)

NF-κB refers to a transcription-factor family that forms protein complexes that regulate DNA transcription in response to diverse cellular stressors, such as inflammation, infection, cytokines and ROS. The spectrum of NF-κB targets is wide. It includes the inflammasome components NLRP3 and caspase-1, the inflammasome substrates pro-interleukin-1 and pro-interleukin-18 [129], adhesion molecules such as intercellular adhesion molecule 1 (ICAM), and tumor necrosis factor-alpha (TNF-α) [130]. The relation between Nrf2 and NF-κB is complex and bi-directional (for review see [96,98]). The Nrf2 gene contains several NF-κB-binding sites, which enable Nrf2 induction by inflammatory stimuli through NF-κB. On the other hand, Nrf2 is able to suppress NF-κB transcriptional activity. The relation between Nrf2 and NF-κB in human CKD has rarely been investigated. The complexity of this relationship is illustrated in a study on Nrf2 and NF-κB gene expression in PBMCs from patients with CKD [131]. Herein, in patients with CKD3/4, higher NF-κB gene expression was associated with higher Nrf2 gene expression, likely reflecting NF-κB-driven pro-inflammatory responses and subsequent Nrf2 activation. On the other hand, in the patient group with CKD5 and hemodialysis therapy, the decrease in Nrf2, which has frequently been observed in advanced CKD [122], was present. In this group, decreased Nrf2 gene expression was associated with significantly increased NF-κB, finally amounting to a doubling of NF-κB gene expression [131] that may reflect a lack of suppressive Nrf2 action on NF-κB. Table 1 provides an overview of the factors involved in endogenous Nrf2 activation in human CKD.

#### 4.1.2. Nrf2 Activation in Patients with CKD According to Cause of CKD, CKD Stage, Comorbidity, and Investigated Cell Type

The assessment of Nrf2 activation and activity in human disease is not straightforward. Nrf2 abundance is regulated on the transcriptional and post-translational levels. Therefore, Nrf2 gene expression, Nrf2 protein amount, Nrf2 protein structure, and Nrf2 transcriptional activity are of relevance [83,96,97,98]. The Nrf2 activation state can be deduced from gene-expression analyses of Nrf2 target genes. Still, also with this approach, some challenges remain. Firstly, Nrf2 transcriptional activity is modified by Nrf2 acetylation, by the availability of MafG, through interaction with the retinoid X receptor (RXR), and by Nrf2’s interaction with CBP [83], which complicates the inference about specific Nrf2-activation modes in cells from a patient. Secondly, for Nrf2 targets such as NQO1 and HO-1, the Nrf2-independent regulation of gene transcription has been reported [132,133,134,135,136,137]. Lastly, from a clinical point of view, it is important whether increased Nrf2-target gene expression in CKD translates into increased target-protein activity, and whether this activity has favorable effects on patient outcomes. In a recent systematic review, the state of Nrf2 and its targets NQO1 and HO-1, from studies reporting data from patients defined as having CKD, were analyzed [122]. Our current report focuses on the activation of the Nrf2 system in patients with different kidney diseases and stages of CKD, as well as resulting therapeutic consequences. To predict the prognosis of CKD, the cause of CKD, GFR category, albuminuria, further risk factors, and comorbidities should be identified [1]. Therefore, we indicate those parameters in relation to the respective Nrf2 characteristics where possible.

##### Nrf2 Activation in Renal Cells of Human CKD

A majority of the analyses in human kidney tissue that are reported below were performed on kidney-biopsy material. A kidney biopsy in a patient is performed for diagnostic reasons, often in the case of thitherto undiagnosed kidney disease or in the case of unexplained worsening of established CKD. It should therefore be noted that the findings for the Nrf2 system in human kidney tissue may frequently reflect a more rapidly progressing disease state.

##### Acute-Kidney-Injury (AKI)-to-CKD Progression

An interesting study investigated kidney biopsies from acute, subacute and chronic tubulointerstitial nephritis [138]. The Nrf2 protein was significantly increased in all types of tubulointerstitial nephritis (TIN), including chronic interstitial nephritis (~CKD3), compared to healthy kidney tissue. However, the highest nuclear and cytoplasmic Nrf2 protein amount was found in acute TIN, thereafter Nrf2 protein gradually decreased to subacute and chronic TIN. Interesting complementary data were provided by a study that compared successful and non-successful renal coping with an AKI event. Successful renal coping was presented by histologically normal kidney-transplant tissue, while non-successful renal coping was represented by kidney tissue from patients with progressive CKD after diverse AKI causes [139]. The time span between the AKI event and the kidney biopsy, comorbidities, or CKD stages were not reported. Compared to healthy kidney tissue, the normal transplant biopsies showed an increase in Nrf2 protein, nuclear Nrf2 accumulation, increased gene expression of Nrf2 target genes NQO1, HO-1 and thioredoxin (Trx1), and concomitantly, a slight increase in oxidative damage. In the example tissues for non-successful renal coping, Nrf2 protein in the cytoplasm showed a pronounced increase compared to healthy and transplanted kidney tissue, but nuclear Nrf2 accumulation seemed diminished, Nrf2-target gene expression was decreased, and oxidative damage was increased. The discrepancy between high Nrf2 protein abundance and decreased Nrf2-target induction was suggested to result from high concomitant tubular GSK-3β protein abundance. While different GSK-3β–mediated mechanisms of Nrf2 repression are known [97], the implications of these mechanisms in human kidney disease have not been sufficiently investigated so far.

##### Diabetes Mellitus and DKD

In an analysis of nephrectomy specimens from patients with diabetes, Nrf2 protein was increased, while Keap1 protein was equal compared to samples from patients without diabetes and normal kidney function [128]. The diabetic population sample size was small and contained patients with no CKD and CKD2–3b with varying degrees of proteinuria. Another study included patients with DKD and proteinuria, but CKD stage was not reported. In this study, Nrf2 and NQO1 protein abundance was increased in diabetic glomeruli [54]. One research group investigated the role of tubular-iron deposition in human CKD [127]. Patients who showed tubular-iron deposition also showed increased protein amounts of ferritin and HO-1, which are both Nrf2 targets [83]. Increased amounts of HO-1 were found in diabetic kidney disease with and without tubular-iron deposition in this study, but the extent of GFR reduction was not reported [127]. A recent publication investigated Nrf2 protein in kidney tissue from different kidney diseases. Diabetic-kidney-disease samples showed increased Nrf2 protein abundance in podocytes and increased Nrf2 nuclear translocation; details on CKD status were not reported [37]. In another small study including patients diagnosed with DKD, on the contrary, a decrease in Nrf2 protein compared to healthy kidney tissue was found [140]. Patient characteristics, CKD stage, or the extent of proteinuria were not reported. In view of partially conflicting results on Nrf2, it is interesting to have a look at one more Nrf2 target protein. Glyoxalase 1 (GLO1) metabolizes cytosolic methylglyoxal, and Nrf2 increases GLO1 expression [141]. In patients with a long duration of diabetes (≥50 years) without the development of DKD, GLO1 protein was significantly increased compared to both age-matched non-diabetic controls without kidney disease and T2D patients (patients with diabetes mellitus type 2) who had developed DKD (CKD3/4) [142]. This could point to a protective role of this Nrf2 target in those patients, who successfully sustained GLO1 upregulation over time.

##### Lupus Nephritis

A larger study on Lupus-nephritis (LN) kidney biopsies reported a significant increase in Nrf2 and NQO1 protein in the LN glomeruli compared to normal kidney tissue [116]. Patients presented with normal to mildly decreased GFR (CKD1/2) but pronounced proteinuria. It is noteworthy that the expression pattern of the Nrf2 protein and its target NQO1 seemingly differed with respect to the LN stages. While the Nrf2 protein increased from stage I to III and decreased again thereafter, the NQO1 protein increased from stage I to V, pointing to differential Nrf2-system-related mechanisms being activated in different LN stages. The previously mentioned study on tubular-iron deposition also investigated LN kidney tissue [127]. The LN samples showed a pronounced increase in tubular HO-1 protein, coincident with tubular-iron deposition.

##### Nrf2 Repression in Renal Cells of Human CKD

Repression of the Nrf2 system in renal cells in human CKD for some etiologies has been described. As noted for several of the publications in the earlier sections, publications on Nrf2 repression also partially lack detailed reporting of patient characteristics such as CKD stage, proteinuria, or comorbidities. For patients with obesity-related nephropathy (~CKD3) a reduction in the renal Nrf2 gene and protein expression was reported in comparison to kidney-tissue samples from patients with nephritis and without GFR reduction [143]. An investigation of autosomal-dominant polycystic kidney disease (ADPKD) reported a significant reduction in Nrf2 protein in ADPKD kidney tissue (~CKD1–3b) compared to healthy controls. Herein, a lower Nrf2 protein amount correlated with a higher total kidney volume and lower GFR, both signs of ADPKD severity [144]. A study on differential gene expression compared calcineurin inhibitor nephrotoxicity (CNIT) in kidney-transplant tissue to normal kidney-allograft tissue. This study identified a large number of Nrf2 targets with decreased gene expression in CNIT [145]. These included 1.6- to 2.0-fold decreases in the gene expression of Trx1, peroxiredoxin, ATP-binding cassette/subfamily C, glutathione S-transferase class M4, microsomal glutathione S-transferase 2, aldehyde dehydrogenase 1 family/member A1, and transaldolase (compare [83,145]). In this respect, it is interesting that another study on kidney-allograft tissue reported increased GSK-3β protein in chronic kidney-allograft dysfunction compared to healthy kidney tissue. Furthermore, GSK-3β expression increased with the severity of tubulointerstitial damage in the transplanted kidneys [146].

Finally, it should be noted that a study that otherwise reported increased Nrf2 protein in membranous nephropathy, fibrillary glomerulonephritis, focal segmental glomerulosclerosis, and diabetic nephropathy did not observe such an increase in renal amyloidosis [37].

##### Nrf2 Status in Non-Renal Cells of Human CKD

Knowledge about Nrf2-system alterations in non-renal cells in CKD is crucial for several reasons. First, circulating cells of the monocyte–macrophage lineage contribute to the pathogenesis of the AKI-to-CKD transition and CKD progression [147,148]. Second, non-renal tissues such as skeletal muscle or cardiovascular tissues are important targets of uremia and are related to survival in CKD [103,149,150]. Third, pharmacological Nrf2 activation targets renal and non-renal cells alike [151].

*Diabetes mellitus and DKD:* One study investigated the Nrf2 target HO-1 in PBMCs from patients with diabetes type 2 with (~CKD1–3) and without DKD [125]. The HO-1 protein was approximately two-times increased in both patient groups compared to healthy control subjects.

*CKD5 with hemodialysis treatment (CKD5-HD):* A multitude of studies on non-renal cells were performed in patients with CKD5-HD. On the one hand, kidney biopsies are usually not performed in those patients. They receive chronic renal-replacement therapy in the end-stage of a kidney disease where kidney function cannot be recovered. On the other hand, there is a constant research interest stimulated by the contribution of non-renal cells to the very high cardiovascular risk, strongly impaired immunity, and high mortality in this patient population. An investigation of synovial vessels reported increased HO-1 protein staining in CKD5-HD compared to a control group with similar comorbidities [117]. A comparable HO-1 gene expression was found between CKD5-HD and a control group with mixed comorbidity conditions including diabetes mellitus when muscle biopsies were analyzed [152]. A third study investigated this Nrf2 target in PBMCs and found a comparable HO-1 gene expression between CKD5-HD and healthy control subjects of comparable ages [153]. The same study also investigated Nrf2 itself and one more Nrf2 target, NQO1. The Nrf2 protein amount and gene expression in CKD5-HD were significantly decreased, as was NQO1 gene expression. Two more studies reported reductions in Nrf2-system components in CKD5-HD mononuclear cells. One study found significantly decreased Nrf2 gene expression compared to both healthy controls and patients with non-dialysis-dependent CKD [131]. The other study reported lower NQO1 gene expression in CKD5-HD than in non-dialysis-dependent CKD [154]. Factors that are relevant for the repression of Nrf2 in human CKD are listed in Table 2.

*Non-dialysis-dependent CKD:* In this patient group, Nrf2 repression in non-renal cells is less frequently reported compared to CKD5-HD. In one investigation of muscle tissue in CKD3b-5, Nrf2 gene expression was decreased compared to healthy control subjects [155]. In PBMCs from patients with CKD (~CKD3–4/5), Nrf2 gene expression was not different from the healthy control group [131], and in patients with DKD (~CKD1–3), the HO-1 protein was significantly increased compared to healthy controls [125]. Finally, compared to healthy subjects, the expression of the Nrf2 target gene NQO1 was significantly increased in CKD1–5 in monocytes [154].

Taken together, the state of the Nrf2 system in human CKD is far from homogenous. It varies, among others, with cause of kidney disease, comorbidities, stage of CKD, duration of CKD, and severity of uremic toxin accumulation and inflammation. Overall, an earlier CKD stage or rapid progression of kidney disease, as well as the inflammatory nature of the underlying kidney disease or comorbidities, were associated with more robust Nrf2-system activation. More advanced CKD, on the other hand, was associated with Nrf2-system repression either in comparison to the healthy condition or to the upregulated state in matched controls. It should be noted that the responsible factors for both the endogenous activation and repression of the Nrf2 system in human CKD have been insufficiently investigated.

The knowledge about the consequences of the state of the Nrf2 system in a certain CKD context is likewise fragmentary. The endogenous activation of the Nrf2 system, as described in DKD or LN, was accompanied by signs of oxidative damage [54,116,117]; therefore, it seems to have been only partially effective. On the other hand, the effective endogenous upregulation of the Nrf2 system has been reported to prevent DKD development in diabetes [142].

### 4.2. Pharmacological Nrf2 Activation in Human CKD

Pharmacological Nrf2 activators that were tested in human CKD to date for therapeutic purposes are electrophilic compounds that target Keap1. This group comprises bardoxolone methyl, curcumin, resveratrol and sulforaphane. They modify Keap1-cysteine-151 and thereby act as Keap1 inhibitors, resulting in the escape of newly synthesized Nrf2 from ubiquitination and degradation [157]. As chronic inflammatory processes and oxidative stress are characteristics of CKD, the hope is that Nrf2 activation could alleviate those features, thereby slowing CKD progression and/or reducing CKD-attributable morbidity. As discussed earlier, the activity state of the Nrf2 system in CKD varies between increased and repressed, dependent on CKD stage, cause of kidney disease, and comorbidities.

Besides the careful analysis of adverse effects, two aspects are of importance with respect to the general prospects of any type of future pharmacological Nrf2-activation therapy in CKD. First, can the respective substance elicit an Nrf2 activation that results in an appropriate functional response of Nrf2 targets in the respective CKD setting? Second, is this response coupled with a desirable effect on CKD progression or a reduction in CKD-attributable morbidity?

We report data on those two aspects for bardoxolone methyl, curcumin, resveratrol and sulforaphane below.

#### 4.2.1. Bardoxolone Methyl

A short history: From 2006 to 2008, bardoxolone methyl was advanced into the first phase I clinical trial as a potential anticancer drug [151]. In this study, an improvement of eGFR was observed that seemed more pronounced in patients with reduced baseline eGFR. A phase II trial in patients with CKD3b-4 and type 2 diabetes (*BEAM* study) found an increase in eGFR of ~6 to 11 mL/min/1.73 m^2^ within 52 weeks of treatment, and an improvement of ~0.7 to 2.5 mL/min/1.73 m^2^ was maintained over four weeks after the completion of dosing [158]. A phase III trial in patients with CKD4 and type 2 diabetes (*BEACON* study) was prematurely terminated due to an increased rate of cardiovascular events [35]. Post-hoc analyses suggested a possible delay of the onset of the end-stage of CKD [159]. One more phase II trial was performed in patients with CKD3–4 and type II diabetes (*TSUBAKI* study). This trial directly evaluated GFR by measuring inulin clearance and found an ~6 mL/min/1.73 m^2^ increase in GFR during the four-month study period [160]. Subsequently, a phase III trial in patients with CKD3–4 caused by DKD was initiated (NCT03550443, *AYAME* study) with an estimated study-completion date in 2024. Additionally, a phase II/III trial was performed in CKD patients with Alport syndrome (NTC03019185, *CARDINAL* study), whose results have not been published or peer-reviewed yet.

The first question concerning the effective activation of Nrf2-system components by bardoxolone methyl in CKD has been insufficiently investigated to date. The initial phase I trial reported a ~2.6-fold increase in NQO1 gene expression on day 2 and a ~5.6-fold increase on day 22 at different bardoxolone-methyl dose levels in PBMCs [151]. Although some of the participants in this analysis might have had reduced kidney function, the exact number is not known.

Serum analyses in the *BEACON* study included the determination of gamma-glutamyl transferase (GGT) [161]. The enzyme GGT, which is a biomarker for liver pathologies, increases in response to oxidative stress and glutathione depletion. It is a biomarker for cardiovascular and metabolic risk [162]. For GGT1, a function as an Nrf2 target has been shown [83], with increased cellular GGT1 gene expression induced by sulforaphane treatment or Keap1 knockdown [163]. Although it needs to be emphasized that the relation between cellular and circulating GGT is not known, the serum GGT data from the *BEACON* study are interesting. The GGT concentration increased steeply after bardoxolone-treatment initiation, reaching a ~2.7-fold increase at the first (day 28) measurement. It remained around this concentration until week 12 and declined slowly thereafter, seemingly converging to a 1.5-fold increase at the last blood sampling in the treatment period at week 48 [161]. The temporal pattern of GGT concentration in these patients with CKD4 could indicate a strong initial Nrf2 activation with a subsequent decline in the effect and convergence to a new “higher-than-baseline” steady state of Nrf2 activation.

The second question of whether the Nrf2 response to bardoxolone methyl is coupled with a desirable effect on CKD progression or a reduction in CKD-attributable morbidity is speculative as long as the molecular Nrf2 responses to bardoxolone in human CKD are not defined. The increase in GFR during and after bardoxolone treatment, its effect size and temporal pattern, possible underlying mechanisms, and whether it indicates a reduction in CKD progression, have extensively been discussed [159,164,165,166] and shall be discussed further when the data from currently finalized or ongoing trials become published. Other effects that were observed in CKD patients during bardoxolone treatment included worsening of pre-existing heart failure, increase in blood pressure/pulse pressure, hypomagnesemia, increased proteinuria, and weight loss. The *TSUBAKI* study also reported an increase in viral upper-respiratory-tract infections. The broad effect spectrum is not surprising, given the central role of Nrf2 in redox, protein and metabolic homeostasis as well as immune responses, and again considering the broad functional spectrum of Nrf2 targets such as NQO1 or HO-1 [96,99,120,167]. The comprehensive investigation of molecular and physiological responses that are/were elicited in this patient population during bardoxolone treatment is urgent.

#### 4.2.2. Sulforaphane

Sulforaphane was first isolated from red cabbage and later from broccoli [168]. Glucoraphanin, the biogenic precursor of sulforaphane, is found in market-stage broccoli, but most abundantly in broccoli sprouts and seeds. A comprehensive review recently discussed issues of sulforaphane sources and dosing requirements [168].

Clinical studies that investigated the regulation of molecular Nrf2 targets in response to sulforaphane/glucoraphanin treatment are sparse. One study on healthy subjects reported an increase in the serum enzyme activity of two Nrf2 targets 24 h after glucoraphanin (NQO1 ~1.3 fold, GST ~1.9 fold). Our review did not identify sulforaphane/glucoraphanin-intervention studies in patients with CKD.

#### 4.2.3. Resveratrol

Resveratrol is a polyphenol found, for example, in grapes and raspberries. Like the aforementioned substances, it targets Keap1, thereby resulting in increased Nrf2 protein. To date, just one study has investigated resveratrol effects on the Nrf2 system in human CKD. Patients with CKD3/4 received resveratrol in a randomized controlled trial. The Nrf2 gene expression was analyzed, and no significant effects were observed [169]. With respect to the anticipated effect of resveratrol on the Nrf2 protein and its activity, an additional investigation of Nrf2 target genes would be of importance. Nevertheless, as Nrf2 can target the Keap1 and the Nrf2 gene itself [83], and since resveratrol has a broad spectrum of further molecular effects [170], Nrf2 gene expression is also a potential target parameter for resveratrol.

#### 4.2.4. Curcumin

Curcumin is a polyphenol compound found in the rhizomes of turmeric (*Curcuma longa*). Curcuminoids account for only around 1 to 6 percent of Curcuma longa extracts, and curcumin is one of those curcuminoids [171].

Our first question, concerning the effective activation of Nrf2-system components by curcumin in CKD, was analyzed in a few studies. One study investigated a mixed population of patients with T2D with and without DKD in an uncontrolled intervention study [172]. After 15 days of curcumin treatment, the study showed in PBMCs a significant increase in Nrf2 (~1.8 fold) and NQO1 (~2.3 fold) protein. The effect occurred in non-DKD and DKD (~CKD1–3A) patients, although the effect was numerically lower in DKD. Two further studies with turmeric in CKD5-HD [173] and CKD3 with and without diabetes [174] did not report significant changes in Nrf2, although opposing directions for Nrf2 changes were observed between the intervention and placebo groups in both studies. The number of patients in both studies was small; well-powered studies are necessary to obtain a meaningful evaluation of the effects on the Nrf2 system. Whether the Nrf2 repression in advanced CKD, as in CKD5-HD, can be overcome by electrophilic compounds is unclear, since endogenous stimulation is already pronounced in this patient group.

The answer to the second question of whether the Nrf2 response to curcumin is coupled with a desirable effect on CKD progression or a reduction in CKD-attributable morbidity is unclear, partially due to the very low number of included CKD patients, and partially because the observed salutary effects were not paralleled by respective Nrf2-system changes. The above-named study on CKD5-HD observed a reduction in inflammatory parameters in the curcumin group (decreased NF-κB gene expression in PBMCs and serum high-sensitivity C-reactive protein (hsCRP)). Another controlled trial in CKD5-HD reported a significant reduction in hsCRP after turmeric treatment for two months [175]. In the abovementioned study on early DKD (CKD1–3A), the increases in Nrf2 and NQO1 were seemingly paralleled by a reduction in albuminuria [172]. Additionally, a controlled study on patients with T2D and early DKD with non-reduced GFR and proteinuria ≥500 mg/d reported decreased urinary protein excretion in patients treated with turmeric for two months [176].

As for sulforaphane, the issue of the source and dosing in curcumin therapy has not been resolved. The discussed studies used curcumin doses between 65 and 500 mg/d. High doses of curcumin (4000–6000 mg/d) administered perioperatively in aortic aneurysm repair increased the risk for acute kidney injury [177].

The issue of curcumin excretion and thereby the possibility for accumulation in human CKD is not clear. Generally, methylation, sulfation and glucuronidation of polyphenols have been described in humans [178]. While it is accepted that urinary excretion of curcumin occurs through glucuronide and sulfate conjugates, the overall proportion of renal excretion in humans is not known [171]. If turmeric is used, the contribution of curcumin to a certain molecular effect is unclear, as turmeric contains a multitude of potentially bioactive compounds [179]. Furthermore, it should be noted that turmeric contains a high percentage of water-soluble oxalate, and turmeric doses comparable to those used in some of the studies (~3 g/d) result in a significantly increased urinary oxalate excretion in healthy subjects [180]. This should raise some caution, as oxalate accumulates in CKD5-HD and high oxalate concentrations in those patients were associated with cardiovascular events [181]. Table 3 summarizes the molecular responses to pharmacological Nrf2 activation reported in human CKD. For comparison, Table 4 summarizes examples of molecular responses to pharmacological Nrf2 activation in non-CKD patients/models or cells.

### 4.3. Future Directions in Nrf2-Targeted Therapies in Human CKD

Successful Nrf2-targeted therapies in CKD require a diversified approach. The Nrf2 system is a complex network that works to ensure adequate responses to redox perturbations, varying energy and metabolic demands, inflammation, and other cellular stresses. This includes dynamic increases and decreases in Nrf2 activity according to demand. The following strategies can be considered:

#### 4.3.1. Targeting Nrf2-System Disturbances in CKD More Specifically

The antioxidant effectiveness through the Nrf2 system in CKD seems to be insufficient in those instances where oxidative damage develops through ROS. The underlying disturbances can occur on all levels of the Nrf2 system, such as inadequate Nrf2 gene expression, Nrf2 protein amount and structure, modulation of Nrf2 transcriptional activity by other factors, and disturbances of Nrf2 targets’ protein amount, structure and activity. Therefore, knowledge about such disturbances in a specific CKD setting is inevitable. If, for example, as reported for chronic kidney-allograft dysfunction, an increase in GSK-3β protein exists [146], then it might be useful to target this kinase using a GSK-3 inhibitor to prevent undue β-TrCP-GSK-3β-mediated Nrf2 degradation. If, as reported in patients with liver cirrhosis, the E3 ubiquitin ligase synoviolin (HRD1) is increased, leading to Nrf2 ubiquitylation and Keap1-independent Nrf2 degradation, then targeting HRD1 rather than Keap1 might be promising [191]. Finally, if already significantly increased NQO1 gene expression, as shown in some CKD stages, does not translate into a correspondingly large increase in NQO1 protein amount [154], then it may be promising to find and target the mechanism responsible for this discrepancy. Altogether, strategies to remove CKD-specific disturbances of the Nrf2 system could result in an improved Nrf2 response with the ability to adequately react to demand.

#### 4.3.2. Pharmacological Keap1 Inhibition

Respective substances, such as bardoxolone methyl, mimic the effects of endogenous electrophiles and oxidants on Nrf2 by inhibiting Nrf2 protein degradation. Thereby, they activate Nrf2-dependent responses of both positively and negatively regulated Nrf2 target genes. Several aspects require attention in this respect. First, is the responsiveness of the Nrf2 system to concentration changes of electrophiles/oxidants in CKD per se preserved? The answer seems to be yes, at least in some patient populations, based on the GGT increase following bardoxolone methyl in CKD4 [161] and the NQO1, HO-1 and ferritin increase following tin protoporphyrin in CKD3/4 [115]. Second, is pharmacological Keap1 inhibition more effective than endogenous Nrf2 activation according to antioxidant requirements? It could be more effective if exhaustive Nrf2 activation is exerted. Third, if the antioxidant demand is permanently increased in CKD, this would require a permanent pronounced Nrf2 activation. Can this be beneficial? The answer is unclear. Endogenous Nrf2 activation is correlated with proteinuria [125], and a higher CVD prevalence was observed in patients with higher NQO1 gene expression [154]. Of course, this does not prove causality. Furthermore, pharmacological Nrf2 activation by bardoxolone methyl led to weight loss at all doses of the BEAM study, the BEACON study, and the TSUBAKI study [85,158,160]. Weight loss is not necessarily negative and was reported to be associated with improved glycemic control in obese patients with T2D [85]. Nevertheless, this effect needs careful observation with respect to patient subgroups, such as normal- or underweight patients or children, and a close observation of the long-term course. This is more important, as there is a high probability that this is a genuine Nrf2-mediated effect. Enzymes that upregulate beta-oxidation, such as carnitine palmitoyltransferase, CD36, and the downregulation of enzymes that induce lipid biosynthesis, such as acetyl-CoA carboxylase, the enzyme that catalyzes lipid biosynthesis, SCD1 and SREBP-1 are regulated by Nrf2 in the respective directions [83]. Finally, based on the reported decline in GGT concentration after the initially strong response to bardoxolone treatment, a restrain of Nrf2 activation through feedback regulation seems likely. Therefore, intermittent dosing schemes, maybe with lower doses, could be worth considering.

#### 4.3.3. Reduction in Factors Responsible for Endogenous Nrf2 Activation

As outlined earlier, a multitude of factors can result in endogenous activation of the Nrf2 system; some of them, as discussed for indoxyl sulfate, may also contribute to Nrf2 repression in high concentrations. To reduce such repression, and to avoid a permanent overactivation of the Nrf2 system, a reduction in Nrf2-stimulating factors in addition to the above proposed strategies is desirable. Those measures will vary according to CKD cause, stage, comorbidities or treatment modality. Indoxyl-sulfate-reducing strategies, for example, comprise oral adsorbents, synbiotics, or special hemodialysis cartridges [192]. Methylglyoxal reduction can be achieved by increasing removal through extended hemodialysis or hemodiafiltration [193], but also through Nrf2 activation by *trans*-resveratrol-hesperitin (tRES-HESP), which induces significant glyoxalase-1 activity and a reduction in methylglyoxal [123].

## 5. Conclusions

Alterations in redox signaling, oxidative stress and disturbed activity of the Nrf2 system have a central role in CKD progression and CKD-related morbidity. The activation of the Nrf2 system in CKD is in multiple ways related to inflammation, kidney fibrosis, and mitochondrial and metabolic effects. Dependent on the actual antioxidant requirements and the disease-related state of other signaling pathways, Nrf2-system activation can be beneficial, but also disadvantageous, depending on the disease and patient context. The Nrf2 system comprises a complex network that functions to ensure adequate responses to redox perturbations, varying energy and metabolic demands, and cellular stresses. It must be kept under homeostatic control within a physiologic activity range. A constant overactivation seems undesirable. It is therefore important to realize that in human CKD, both endogenous Nrf2 activation and repression exist. The state of the Nrf2 system varies with cause of kidney disease, comorbidities, stage of CKD, duration of CKD, and severity of uremic toxin accumulation and inflammation. An earlier CKD stage or rapid progression of kidney disease, as well as inflammatory processes, are associated with a more robust Nrf2-system activation. Advanced CKD is associated with stronger Nrf2-system repression. For the development and evaluation of future Nrf2-targeted therapies, it is necessary to answer the following questions: Is the substance able to elicit an Nrf2 activation that results in an appropriate functional response of Nrf2 targets in the respective CKD setting? Is this response coupled with a desirable effect on CKD progression or a reduction in CKD-attributable morbidity? Is the substance overall beneficial for the patient? Therefore, future Nrf2-targeted therapies in CKD should apply a diversified approach that enables dynamic increases and decreases in Nrf2 activity according to homeostatic requirements. The resulting Nrf2-system responses should be sufficient to cope with oxidative stress, inflammatory state, and activated pro-fibrotic mechanisms. Such new approaches need to be fitted to specific CKD-related disturbances in the Nrf2 system, such as increases in GSK-3β, CKD-related protein modifications on Nrf2 and Nrf2 target proteins, and accumulation of uremic toxins. While the aim is to support Nrf2 activity, Nrf2 overactivation should be avoided.

## Figures and Tables

**Table 1:** Factors and mechanisms relevant for endogenous Nrf2 activation in human CKD.

Factor	Possible Mechanism	Observed Effects in Patients with CKD	Reference for the Patient Data
Oxidative stress	Oxidants and thiol-reactive electrophiles modify Keap1 → increase in Nrf2 translocation to the nucleus → effect on transcription of Nrf2 targets	DKD: oxidative damage of renal glomeruli,	[54]
		Nrf2 protein ↑	[116]
		NQO1 protein ↑	
		LN: oxidative damage of renal glomeruli,	
		Nrf2 protein ↑	
		NQO1 protein ↑	
		CKD5-HD: synovial tissue	[117]
		MDA ↑	
		HO-1 protein ↑	
Uremic toxins Indoxyl sulfate	(a) induction of ROS → effect on transcription of Nrf2 targets as above (b) activation of AhR → induction of Nrf2 gene transcription	CKD3/4:PBMCs, positive correlation between plasma indoxyl sulfate (1–11 mg/L) and Nrf2 gene expression	[121]
Methylglyoxal	Keap1 cross-linking → Keap1 dimers → Nrf2 accumulation and Nrf2-target induction	T2D with and without DKD: Lymphocytes, HO-1 protein ↑ Plasma, NQO1 protein ↑ Renal cells, Nrf2, NQO1, HO-1 protein ↑	[125]
			[126]
			[37,54,127,128]
NF-κB	NF-κB binding sites in Nrf2 gene → Nrf2 induction	CKD3/4: PBMCs, positive correlation between NF-κB and Nrf2 gene expression ↑	[131]

Abbreviations: Keap1—Kelch-like ECH-associated protein 1; ROS—reactive oxygen species; NF-κB—nuclear factor kappa B; Nrf2—Nuclear factor erythroid 2-related factor 2; AhR—arylhydrocarbon receptor; DKD—diabetic kidney disease; NQO1—NAD(P)H:quinoneoxidoreductase 1; LN—Lupus nephritis; CKD—chronic kidney disease; MDA—malondialdehyde; HO-1—heme oxygenase 1; PBMC—peripheral blood mononuclear cell; T2D—Diabetes mellitus type 2; →—leading to, ↑—increased.

**Table 2:** Factors and mechanisms relevant for endogenous Nrf2 repression in human CKD.

Factor	Possible Mechanism	Observed Effects in Patients with CKD	Reference for the Patient Data
Increased GSK-3β	GSK-3β → phosphorylation of Nrf2 → ubiquitinylation by β-TrCP → Nrf2 degradation ↑	ADPKD_CKD1–3b: kidney tissue, Nrf2 protein ↓	[144] *
Uremic toxins Indoxyl sulfate	↓ Nrf2 gene and protein expression with high indoxyl-sulfate concentrations	CKD-5HD: PBMCs, plasma indoxyl sulfate (mean ~29 mg/L) correlated negatively with Nrf2 gene expression	[156]

* The increase in GSK-3β was only shown in the ADPKD mouse model in this publication. Abbreviations: GSK-3β—glycogen synthase kinase 3; Nrf2—Nuclear factor erythroid 2-related factor 2; β-TrCP—F-box/WD repeat-containing protein 1A; ADPKD—Autosomal dominant polycystic kidney disease; CKD—chronic kidney disease; HD—hemodialysis, PBMC—peripheral blood mononuclear cell; →—leading to, ↑—increased; ↓—decreased.

**Table 3:** Molecular responsiveness of the Nrf2 system to pharmacological Nrf2 activation in human CKD with respect to CKD stage and comorbidity.

Substance	Nrf2-System Response	Reference
Bardoxolone methyl	CKD4 and T2D: serum GGT ↑	[161]
Resveratrol	CKD3/4: no effect on PBMC Nrf2 gene expression	[169]
Curcumin	CKD1–3a and T2D: PBMC Nrf2 and NQO1 protein ↑	[172]
Curcumin	CKD5-HD, diabetes, hypertension: no effect on PBMC Nrf2 gene expression	[173]
Curcumin	CKD, proteinuria, diabetes: no effect on PBMC Nrf2 binding activity	[174]
Tin proto-porphyrin	CKD3/4, diabetes, hypertension: plasma NQO1, HO-1, and ferritin ↑	[115]

Abbreviations: CKD—chronic kidney disease; T2D—Diabetes mellitus type 2; GGT—gamma-glutamyl transferase; PBMC—peripheral blood mononuclear cell; T2D—Diabetes mellitus type 2; Nrf2—Nuclear factor erythroid 2-related factor 2; NQO1—NAD(P)H:quinoneoxidoreductase 1; HO-1—heme oxygenase 1; ↑—increased.

**Table 4:** Examples of molecular responses to pharmacological Nrf2 activation in non-CKD patients/models or cells.

Substance	Response	Reference
Curcumin	NQO1 activity↑	[182]
Curcumin	HO-1 protein↑	[183]
Curcumin	GLO1 activity↓	[184]
Resveratrol	NQO1 protein↑	[185]
Resveratrol	HO-1 protein↑	[185]
Resveratrol	GLO1 gene expression and activity↑	[186]
Sulforaphane	NQO1 activity↑	[187]
Sulforaphane	HO-1 protein↑	[188]
Sulforaphane	GLO1 protein and activity↑	[189]
Bardoxolone methyl	NQO1 gene expression↑	[34]
Bardoxolone methyl	HO-1 gene expression and protein↑	[190]

Abbreviations: NQO1—NAD(P)H:quinoneoxidoreductase 1; HO-1—heme oxygenase 1; GLO1—glyoxalase 1; ↑—increased; ↓—decreased.

## Supplementary Materials

The following supporting information can be downloaded at: https://www.mdpi.com/article/10.3390/antiox11061112/s1. Table S1: Abbreviations; Supplementary material S1: Overview of the literature search strategy used for this narrative review.
