# Supplementary material for: Nrf2 Activation in Chronic Kidney Disease: Promises and Pitfalls
Source: Antioxidants (Basel). 2022 Jun 3;11(6):1112. doi: 10.3390/antiox11061112 (PMC9220138; doi:10.3390/antiox11061112)
Supplement: Supplementary file 1 [file antioxidants-11-01112-s001.zip › antioxidants-1747691-supplementary.pdf]

Supplementary table S1. Abbreviations.

Abbreviation	Full form
8-Oxo-dG	8-hydroxy-2'-deoxyguanosine
ACC1	acetyl-CoA carboxylase
ACE	angiotensin-converting enzyme
ADPKD	autosomal dominant polycystic kidney disease
AGEs	advanced glycation end products
AhR	arylhydrocarbon receptor
AKI	acute kidney injury
Akt	protein kinase B
AMPK	5' AMP-activated protein kinase
Ang II	angiotensin II
ARE	antioxidant response element
ASC	apoptosis-associated speck-like protein containing a CARD (caspase activation and recruitment domain)
ATP5A	ATP synthase 5A
bZip	basic leucine zipper
CBP	cyclic adenosine monophosphate (cAMP) response element-binding protein (CREB)-binding protein
CKD	chronic kidney disease
COX	cyclooxygenase
CPT1	carnitine palmitoyl transferase-1
CUK	α-cyano-α, β-unsaturated ketone
Cul3	cullin3
CVD	cardiovascular disease
Cys	cysteine
DKD	diabetic kideny disease
DN	diabetic nephropathy
EMT	epithelial-mesenchymal transition
GCLC	glutamate-cysteine ligase catalytic
GCLM	glutamate-cysteine ligase modifier
GFR	glomerular filtration rate
GGT	gamma-glutamyl transferase
GLO1	glyoxalase 1
GPx	glutathione peroxidase
GSK3	glycogen synthase kinase 3
GST	glutathione S-transferase
H_2O_2	hydrogen peroxide
HD	hemodialysis
HK-2	human kidney 2
HO-1	heme oxygenase 1
HRD1	E3 ubiquitin ligase synoviolin
hsCRP	high-sensitivity C-reactive protein
ICAM	intercellular adhesion molecule 1

IL	interleukin
IR	ionizing radiation
IκB	nuclear factor of kappa light polypeptide gene enhancer in B-cells inhibitor
Keap1	Kelch-like ECH-associated protein 1
LLCPK	Lilly Laboratories Culture-Porcine Kidney 1
LN	lupus nephritis
Maf	musculoaponeurotic fibrosarcoma
MAPK	mitogen-activated protein kinase
MCP-1	monocyte chemoattractant protein-1
NF-κB	nuclear factor kappa B
NLRP3	NOD-like receptor family pyrin domain containing 3
NOX	NADPH oxidase
NQO1	NADPH dehydrogenase quinone 1
NRF1	nuclear factor respiratory 1
Nrf2	nuclear factor erythroid 2-related factor 2
oxLDL	oxidized low-density lipoproteins
OXPHOS	oxidative phosphorylation
PBMCs	peripheral blood polymorphonuclear cells
PGC-1α	peroxisome proliferator-activated receptor gamma coactivator 1-alpha
RAS	renin-angiotensin system
Rbx1	ring box 1
ROS	reactive oxygen species
RTA 405	synthetic triterpenoid
RXR	retinoid X receptor
SCD1	stearoyl-CoA desaturase
SFN	sulforaphane
SOD1/2	superoxide dismutase 1/2
SREBP-1	sterol regulatory element-binding protein-1
STZ	streptozocin
T2D	diabetes mellitus type 2
TCA	tricarboxylic acid
TFAM	mitochondrial transcription factor A
TIN	tubulointerstitial nephritis
TNF-α	tumor necrosis factor-alpha
tRES-HESP	trans-resveratrol-hesperitin
Trx1	thioredoxin
UUO	unilateral ureteral obstruction
α-SMA	alpha-smooth muscle actin
β-oxidation	fatty acid beta-oxidation
β-TrCP	F-box/WD repeat-containing protein 1A

Supplementary material S2. Overview of the literature search strategy used for this narrative review.

Search strategy

For the preclinical data of this review, PubMed, Web of Science, and Google scholar databases were examined to find studies on Nrf2 and chronic kidney disease. Databases were searched from January 2022 to March 2022. The final comprehensive search was performed 26.03.2022. In addition, the reference lists of original articles and relevant review articles were hand searched, yielding more relevant publications.

Search terms

Pubmed

((((“NRF2” OR “nuclear factor erythroid 2-related factor 2”)) AND (nephropathy OR “kidney injury” OR “kidney disease“ OR “kidney diseases“ OR “kidney aging“ OR ”end-stage kidney disease“ OR “5/6 nephrectomy” OR diabetic nephropathy OR “diabetic kidney disease” OR “chronic kidney disease” OR “CKD”)) AND AND (Keap1) AND (“oxidative stress OR reactive oxygen species OR ROS) AND (NQO1 and HO-1) AND (sulforaphane OR SFN OR bardoxolone) AND (inflammation) AND (NF-κB) AND (inflammasome) AND (NRLP3) AND (TGF-β1) (fibrosis) AND (RAS) AND (angiotensin-converting enzyme 2 OR ACE2) AND (angiotensin II OR AngII) AND (mitochondria) AND (nuclear factor respiratory 1 OR NRF1) AND (peroxisome proliferator-activated receptor gamma coactivator 1-alpha OR PGC-1α) AND (mitochondrial transcription factor A OR TFAM) AND (beta-oxidation OR β-oxidation) AND (carnitine palmitoyl transferase-1 OR CPT1) AND (stearoyl-CoA desaturase OR SCD1) AND (sterol regulatory element-binding protein-1 OR SREBP-1) AND (cancer resistance OR resistance to cancer therapy OR radiotherapy) AND (atherosclerosis) NOT (humans[MH])

For the clinical data of this review, we searched text words and Medical Subject Heading (MeSH) terms in PubMed between January and March 2022. The final comprehensive search was performed 24.03.2022. Additionally, the reference lists of relevant original articles and review articles were hand-searched for further relevant publications.

Search terms

Pubmed

(((nephritis OR nephropathy OR “kidney injury” OR “renal dysfunction” OR dialysis OR „renal dialysis“ OR hemodialysis OR haemodialysis OR "renal replacement therapy" OR „kidney replacement therapy“ OR CAPD OR APD OR CCPD OR "renal insufficiency" OR „kidney insufficiency“ OR uremia OR uraemia OR uremic OR CKD OR CKF OR CRD OR CRF OR „kidney disease“ OR „kidney diseases“ OR „kidney failure“ OR „renal failure“ OR „chronic renal insufficiency“ OR „chronic kidney insufficiency“ OR ESRD OR ESKD OR ESRF OR ESKF OR „end stage kidney disease“ OR „end-stage kidney disease“ OR „end stage renal disease“ OR „end-stage renal disease“ OR „chronic kidney failure“ OR „chronic renal failure“ OR „end stage kidney failure“ OR „end-stage kidney failure“ OR „end stage renal failure“ OR „end-stage renal failure“ OR „chronic kidney disease“)) AND (“NRF2” OR “nuclear factor erythroid 2-related factor 2”)) NOT (animals[MH] NOT humans[MH])

Eligibility criteria

Studies were included that had investigated NRF2 and/or established NRF2 target genes/proteins or NRF2 targeted therapies in patients with kidney disease. We used the following exclusion criteria: Studies investigating exclusively acute kidney injury (AKI). There was neither a language restriction, nor an age limit for publication date for included studies.
